# Effects of Monotypic and Binary Mixtures of Metal Oxide Nanoparticles on Microbial Growth in Sandy Soil Collected from Artificial Recharge Sites

**DOI:** 10.3390/ijms161126066

**Published:** 2015-11-24

**Authors:** Kyung-Seok Ko, Kyoochul Ha, In Chul Kong

**Affiliations:** 1 Groundwater Department, Geologic Environment Division, Korea Institute of Geoscience and Mineral Resources (KIGAM), Daejeon 34132, Korea; kyungsok@kigam.re.kr (K.-S.K.); hasife@kigam.re.kr (K.H.); 2 Department of Environmental Engineering, Yeungnam University, Gyeongbuk 38541, Korea

**Keywords:** ATP, DHA, nanoparticles, microbial growth, mixture, soil, VCC

## Abstract

The potential effects of monotypic and binary metal oxide nanoparticles (NPs, ZnO, NiO, Co_3_O_4_ and TiO_2_) on microbial growth were evaluated in sandy soil collected from artificial recharge sites. Microbial growth was assessed based on adenosine triphosphate (ATP) content, dehydrogenase activity (DHA), and viable cell counts (VCC). Microbial growth based on ATP content and VCC showed considerable differences depending on NP type and concentration, whereas DHA did not significantly change. In general, ZnO NPs showed the strongest effect on microbial growth in all measurements, showing an EC_50_ value of 10.9 mg/L for ATP content. The ranking (EC_50_) of NPs based on their effect on microbial growth assessed by ATP content and VCC was ZnO > Co_3_O_4_ > NiO > TiO_2_. Upon exposure to binary NP mixtures, synergistic and additive modes of action were observed for ATP content and VCC, respectively. The ranges of observed (P(O)) and expected (P(E)) activity were 83%–92% and 78%–82% of the control (*p*-value 0.0010) based on ATP content and 78%–95% and 72%–94% of the control (*p*-value 0.8813) based on VCC under the tested conditions, respectively. The results indicate that the effects of NP mixtures on microbial growth in the sandy soil matrix were as great, or greater, than those of single NPs. Therefore, understanding the effects of single NPs and NP mixtures is essential for proper ecological risk assessment. Additionally, these findings demonstrate that the evaluation of NP effects may be profoundly influenced by the method of microbial growth measurement.

## 1. Introduction

Engineered artificial recharge systems are achieved by ponding or flowing water onto the soil surface to augment groundwater water resources [1]. The most likely reason for failure of infiltration is clogging. Clogging of artificial recharge systems is generally caused by interdependent mechanisms (physical, chemical, and biological processes). However, clogging likely occurs due to microbiological growth, such as biofilms, on the infiltration surface [2]. Extracellular polysaccharides (EPS), which are key components of the microbial matrix, mediate bacterial adhesion to the surface, provide mechanical stability to the biofilm, and protect cells against external aggression [3]. 

These days, many products from nanotechnology are available in the fields of electronics, optics, food packaging, textiles, medical devices, cosmetics, fuel cells, catalysts, biosensors, and components for environmental treatment [4]. Due to rapid nanotechnology development, nanoparticles (NPs) have been increasingly used for industrial applications and will inevitably enter natural ecosystems, with soil predicted to be a substantial sink [5]. Therefore, the production, use, and disposal of NPs have inevitably led to their release into the environment through atmospheric emissions, domestic wastewater, and agriculture byproducts, either accidentally through manufacturing and transport or intentional release. However, there are a lack of quantitative knowledge and appropriate methods for detecting and quantifying NPs in complex natural media [6]. NPs are generally classified into four categories: (1) carbon-based materials; (2) metal-based materials; (3) dendrimers; and (4) composites [7]. Their unique physicochemical properties (e.g., extremely small size, surface characteristics, reactivity, conductivity, optical sensitivity, *etc.*) are often associated with unique adverse biological effects in ecosystems [8,9,10,11,12]. Regarding NPs of silver, platinum, and carbon nanotubes, there have been several reports of their toxic effects on terrestrial animals and bacteria [13,14]. NPs may exhibit different toxicological effects according to particle variety and size, test organism species, and test method [15].

The environment is generally exposed to complex mixtures of contaminants, but most studies have mainly focused on the effect of single contaminants under laboratory conditions [16]. Therefore, studies on the mixture effects rather than single ones more realistically reflect ecosystem pollution. Unfortunately, predicting the response of an organism by more than one potentially toxic chemical is one of the most difficult matters in environmental risk assessment [17]. In order to solve this problem, mixture models can be used and generally classified into two basic types: concentration addition models and response (effects) addition models. Additionally, the theoretically expected effects of binary mixtures on test organisms can be calculated using a simple mathematical model based on the theory of probabilities [18] or by using the toxic unit (TU) approach [19], which is mostly used to examine mixtures [20,21]. The mixture effect is, thus, defined as being either similar to (additive), greater than (synergistic), or less than (antagonistic) additive in relation to theoretically expected effects calculated based on individual chemicals.

Biomass can be measured by various indirect methods, such as measurement of ATP content and DHA, which are common methods for environmental samples. Cellular ATP is commonly measured based on the production of bioluminescence by a luciferin–luciferase reaction [22]. Dehydrogenase enzyme activity is also an appropriate measure of biological activity and an indirect indicator for environmental samples [23]. In addition, classical heterotrophic plate counting (VCC) is a direct method involving the detachment of microorganisms from the surface and then counting culturable cells on solid media. Recently newly developed techniques are reported for the monitoring of the microbial growth in environmental systems [24,25].

In this study, the inhibitory effects of single and binary mixtures of metal oxide NPs on microbial growth were examined using sandy soil collected from artificial recharge sites. Moreover, the effects of NPs on microbial growth were evaluated using three different methods (ATP, DHA, and VCC) of biomass measurement. 

## 2. Results

### 2.1. Effects of Single NPs on Microbial Growth

**Figure 1:**
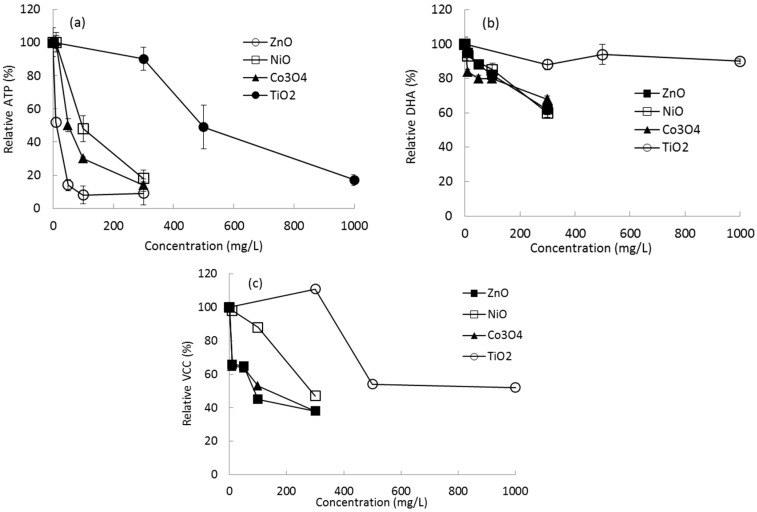
Effects of NPs on microbial growth on sandy soil based on relative (**a**) ATP content; (**b**) DHA; and (**c**) VCC.

After initial definitive tests, different concentration ranges of each type of NP were used to evaluate the effects on microbial growth on sandy soil. Microbial growth was determined using both indirect (ATP content and DHA) and direct (VCC) methods. For a control without NP exposure, the quantities of ATP, DHA, and VCC from microbial growth on the soil surface were in the ranges of 7.13 ± 0.25∼7.75 ± 0.68 ng ATP/g soil, 16.25 ± 0.485∼16.13 ± 0.462 μg/g soil, and 3.28 × 10^7^ CFU/g soil, respectively, depending on the batch set. Profiles of ATP content in the presence of NPs are shown in Figure 1. No stimulation of microbial growth was observed under the tested NP concentrations. Weak or no inhibition was observed at the lowest concentration tested for each NP (10 mg/L of NiO (100% of control), Co_3_O_4_ (101% of control), and 300 mg/L TiO_2_ (90% of control)), whereas considerable inhibition was observed at 10 mg/L of ZnO (52% of control). Relative ATP content and VCC were observed to be in the ranges of 101% (10 mg/L of Co_3_O_4_), −9% (300 mg/L of ZnO), 111% (300 mg/L of TiO_2_), and −38% (300 mg/L of ZnO) of the control, respectively (Figure 1). However, in the case of DHA observation, the highest inhibition was 61% of the control at 300 mg/L of NiO (Figure 1).

Based on these results, the effects of the NPs on microbial growth were evaluated according to their EC_50_ values, which were calculated using the SPERMAN program (Table 1). EC_50_ values for DHA were not feasibly calculated because all EC_50s_ exceeded the tested maximum concentration (>300 mg/L for NiO, ZnO, and Co_3_O_4_; >1000 mg/L for TiO_2_). EC_50_ values based on ATP content were observed to be in the range of 11 mg/L (ZnO) to 530 mg/L (TiO_2_). The ranking of NP inhibition (EC_50_) in terms of either ATP content or VCC was in the same order: ZnO > Co_3_O_4_ > NiO > TiO_2_. Among the tested NPs, ZnO showed the strongest inhibition against microbial growth on sandy soil based on ATP content (EC_50_ of 11 mg/L) or on VCC (EC_50_ of 17.6 mg/L) (Table 1). In terms of ATP content, this value is approximately 48 times less than that of TiO_2_, which showed the weakest inhibition among the tested NPs.

**Table 1:** Comparisons of NP toxicity on microbial growth on sandy soil (95% confidence level).

Methods	Nanoparticle EC_50_ (mg/L)			
	NiO	ZnO	Co_3_O_4_	TiO_2_
ATP	87 (64.6–116.4) **^a^**	11 (7.3–16.3)	55 (44.6–66.1)	530 (479.1–587.1)
DHA	>300	>300	>300	>1000
VCC	277 (216.0–354.8)	17.6 (12.74–24.30)	127 (81.0–198.3)	>1000

**^a^** Value is the range of the 95% confidence level (low limit–high limit).

### 2.2. Effects of Binary NP Mixtures on Microbial Growth

**Figure 2:**
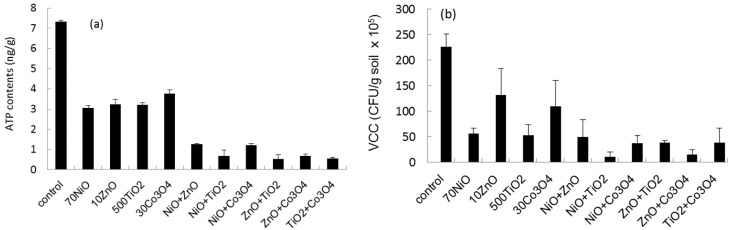
Profiles of (**a**) ATP content and (**b**) VCC in the presence of monotypic and binary mixtures of ZnO, NiO, Co_3_O_4_, and TiO_2_.

The effects of binary mixtures on microbial growth on sandy soil were investigated using four NPs (ZnO, Co_3_O_4_, NiO, and TiO_2_) based on ATP and VCC measurements. Each NP type was tested at one concentration (10 mg/L ZnO, 70 mg/L NiO, 30 mg/L Co_3_O_4_, and 500 mg/L TiO_2_) similar to the EC_50_ value of single NPs based on ATP measurement (Table 2). The ATP content and VCC of sandy soil exposed to single and binary NP mixtures after 30 days are shown in Figure 2. The control (no NP amendment) showed a mean ATP level of approximately 7.3 ± 0.06 ng ATP/g soil after 30 days of incubation, whereas sets amended with single or binary NP mixtures showed ATP levels of 3.1–3.8 and 0.53–1.26 ng ATP/g, respectively, corresponding to 42%–52% and 7.3%–17.3% of the control. In the case of VCC, the single and binary NP mixture values were 23%–58% and 5%–22% of the control, respectively.

**Figure 3:**
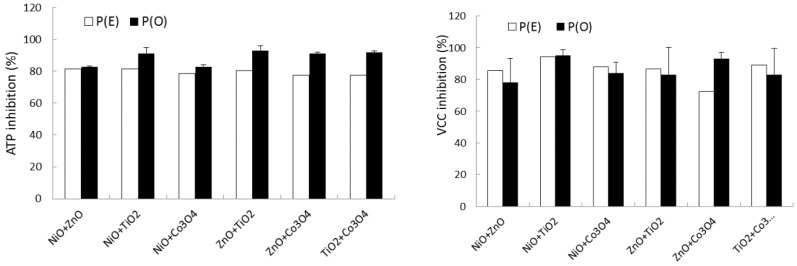
Comparisons of P(O) and P(E) for binary NP mixtures based on ATP content and VCC.

The observed inhibitory effect, P(O), of a binary equal mixture was compared with its expected inhibitory effect, P(E), which was theoretically calculated based on two measurements of single effects using the theory of probabilities (Figure 3). The observed ATP content of the binary mixture was in the range of 8%–17% of the control (no NP amendment), whereas the expected value was in the range of 18%–22% of the control. Comparisons between P(O) and P(E) for the ATP observations are shown in Figure 3. In the case of ATP inhibition, P(O) and P(E) were 83%–93% and 78%–82% of the control, respectively. Statistically significant differences between P(O) and P(E) were observed in ATP content (*p*-value = 0.001). In the case of VCC inhibition, P(O) and P(E) were 78%–93% and 72%–94% of the control (Figure 3). There were no significant differences between P(E) and P(O) (*p*-value = 0.8813). 

Metal ions released from amended NPs in solutions of single and binary NP mixtures were measured after 30 days (Table 2). The concentration (%) of metal ions released from the tested NPs in sandy soil slurry ranged from a maximum of 6% to a minimum of <0.02% of the amended NP concentration. In the case of sets with 10 mg/L of ZnO, 70 mg/L of NiO, 30 mg/L of Co_3_O_4_, and 500 mg/L of TiO_2_, for monotypic NPs or mixtures, the metal ion ranges were as follows: 0.366–0.603 mg Zn/L, 2.024–2.952 mg Ni/L, 0.042–0.082 mg Co/L, and 0.016–0.065 mg Ti/L, corresponding to 3.66%–6.03%, 2.89%–4.22%, 0.14%–0.27%, and 0.003%–0.013%, respectively. 

**Table 2:** Metal ion concentration in sandy soil slurry solution after 30 days of incubation.

NPs (mg/L)	Metal Ion (mg/L)	NPs (mg/L)	Metal Ion (mg/L)
ZnO (10)	0.60 ± 0.065	NiO + TiO_2_ (70 + 500)	2.024/0.054
Co_3_O_4_ (30)	0.04 ± 0.015	NiO + Co_3_O_4_ (70 + 30)	2.524/0.042
NiO (70)	2.95 ± 0.416	ZnO + TiO_2_ (10 + 500)	0.387/0.054
TiO_2_ (500)	0.07 ± 0.016	ZnO + Co_3_O_4_ (10 + 30)	0.616/0.082
NiO + ZnO (70 + 10)	2.23 ± 0.366	TiO_2_ + Co_3_O_4_ (500 + 30)	0.016/0.079

## 3. Discussion

In this investigation, the effects of monotypic NPs and binary NP mixtures on microbial growth in sandy soil were examined after 30 days of incubation. To facilitate the study of bacterial attachment, a variety of direct and indirect experimental observation methods have been developed [26]. ATP is an energy carrier present in all active cells and is used to measure the amount of active biomass. ATP bioluminescence has been proposed as a technique for estimating microbial activity in a biological process since 1960 [27]. In general, classical heterotrophic plate counting (VCC) includes only culturable cells, whereas ATP measurement also includes bacteria that do not contribute to these counts. The ATP method has been criticized due to the wide variance between ATP content and biomass, however it does hold promise due to its small sample requirement, large number of replicates, and rapid rate [27]. Some researchers have reported that standard plate count methods provide an inaccurate estimate of the number of microorganisms in samples and therefore cannot be used in quantitative measurements [28]. DHA could serve as an indirect indicator of microbial activity in various microbial systems [29]. In this investigation, the effects of NPs on microbial growth on sandy soil were evaluated using indirect (ATP content and DHA) and direct (VCC) measurements. 

In a control without NP exposure, ATP, DHA, and VCC in microbial growth on the soil surface were in the ranges of 7.71–12.36 ng ATP/g soil, 17.10–37.35 μg/g soil, and 1.53 × 10^7^–6.28 × 10^7^ CFU/g soil, respectively. Generally, investigators have reported that microorganisms produce 10^−8^ to 10^−9^ μg ATP/cell [27]. Based on this report, the biomass calculated from ATP content was slightly less than that based on VCC. ATP quantities may differ depending on the culture conditions. For example, bacteria in drinking water typically have stable ATP/cell values in the range of 0.7 × 10^−7^–2.3 × 10^−7^ ng ATP/cell [30,31]. Therefore, both results present reasonably similar corresponding quantities of biomass. 

Based on a preliminary test, different concentration ranges of NPs were adopted for the investigation: 10–300 mg/L of ZnO, NiO, and Co_3_O_4_ and 300–1000 mg/L of TiO_2_. In general, no stimulation of microbial growth was observed at the tested NP concentrations. The experimental results showed that ATP content and VCC were significantly different according to NP dose and type but not DHA. In the presence of various concentrations of ZnO, NiO, Co_3_O_4_, and TiO_2_ individually, the highest inhibition of microbial growth was observed at 300 mg/L of ZnO, showing 0.71 ± 0.53 ng of ATP (91% inhibition) and 1.24 × 10^7^ CFU (62% inhibition) per g of sandy soil. Relatively low inhibition of DHA was observed (10%–40% inhibition for all NPs) under all conditions. At the highest tested concentrations (300 mg/L of ZnO, NiO, and Co_3_O_4_ and 1000 mg/L of TiO_2_), all the tested NPs showed significant reduction of microbial growth (82%–91% and 48%–96% relative inhibition of ATP and VCC, respectively). With respect to sensitivity (EC_50_ values), the order of microbial growth inhibition of the NPs based on ATP and VCC was as follows: ZnO > Co_3_O_4_ > NiO > TiO_2_. The EC_50_ values for DHA were all higher than the tested maximum concentration of each NP. In terms of ATP content, the EC_50_ of toxic ZnO was approximately 50-fold lower than that of TiO_2_, which was the least toxic NP. In a previous report on NP toxicity, ZnO also showed higher toxicity than NPs of NiO, Co_3_O_4_, and TiO_2_ in terms of bacterial bioluminescence activity and seed germination [32]. 

No single observation may show uniform inhibition and sensitivity to chemicals. ATP-based observations are used routinely to detect bacteria because ATP concentrations are relatively constant across many different growth conditions [33]. Sule *et al.* [33] reported that an ATP assay, crystal violet assay, and scanning electron microscopy yielded similar results for six of eight strains tested. In this investigation, both ATP and VCC showed considerable sensitivity based on the conditions and type of NP. However, researchers have reported that VCC measurements may have the following limitations: (1) ineffective at removing adhered cells, making counts on agar plates inconsistent with the actual number of bacteria adhered to the surface; (2) some biofilm systems and cultivation conditions may induce cells into a “viable but nonculturable” state in which they are metabolically active but unable to divide and form colonies in culture medium, probably as a survival mechanism in response to adverse conditions [34,35]. The reason why the results for DHA were insensitive compared to those for ATP and VCC is not clear at this point, but enzymatic activity may be dependent on the type of matrix. For example, dehydrogenases and acid phosphatases exhibit greater activity in heavy loamy sand, whereas their activity is not greater in silty sandy loam [23]. In our previous investigation using compost samples, insensitive DHA patterns were also observed for various compost samples, whereas the ATP content of the samples differed considerably depending on the conditions [36]. Therefore, DHA might not correlate well with the biomass content in biofilm because DHA activity can vary depending on the physiological conditions in a microbial system composed of various microbial species [37]. Based on the results from all three assays, a combination of ATP and VCC assays appears to be the most effective quantitative measurement of the effects of NPs on microbial growth on sandy soil. The data obtained from the ATP assays in this study also showed patterns similar to those of total microbial biomass and VCC. 

Although their precise toxicity mechanisms are largely unknown, studies have shown that the toxicity of NPs is generally governed by properties such as particle size, shape, and surface properties [38,39]. Some studies reported that the effects of NPs on bacteria could be occurred by mechanisms, such as membrane disorganization, DNA damage, surface photocatalytic oxidation, and reactive oxygen species (ROS) production [40,41,42,43]. NPs also may undergo agglomeration, sorption, desorption, dissolution, and migration in different soils with different textures, pHs, ionic strengths, and organic matter content. NPs may vary in bioavailability and alter soil microorganisms’ exposure to them [44]. Dhas *et al.* [45] reported that Ag and ZnO NPs disrupt the cell membrane of bacterial cells by making pits in it due to their small size, leading to increased membrane permeability and cell death. Tong *et al.* [46] and Marambio-Jones and Hoek [47] reported that the toxicity of Ag NPs is mainly due to the effects of free ions. On the other hand, TiO_2_ NPs are chemically stable, and their toxicity is predominantly caused by ROS generation with ultraband gap excitation. The antimicrobial activity of NPs may also be related to their positively charged surface. Electrostatic force might contribute greatly to the adhesion of positively charged particles [45]. The antibacterial mechanism of ZnO nanoparticles is most likely due to disruption of the cell membrane, which affects its permeability [48]. Lopes *et al.* [49] reported that dissolution and particle size in daphnia test media were to be essential to NP toxicity. At this point, it is not clear whether the toxicity was induced by the particles or by released ions. In our further investigation, the effect of metal ion concentration (0.6 mg Zn/L of ZnCl_2_ and 2.95 mg Ni/L of NiCl_2_) of 10 mg/L ZnO and 70 mg/L NiO was evaluated based on bacterial bioluminescent activity, which showed approximately 20% and 25% inhibition of control, respectively. Therefore, the toxicity might be induced both by the particles and by released ion concentration, which will be depended on tested conditions. 

The effects of binary mixtures of NPs on microbial growth were investigated based on ATP and VCC measurements. A binary mixture may show three different effects, such as antagonistic (less than additive), synergistic (greater than additive), and additive effects [50]. The activity of the binary NP mixtures was in the range of 7%–17% and 5%–22% of the control based on ATP content and VCC, respectively. Expected mixture effects were determined based on the theory of probabilities [18]. Based on the theory of probabilities, statistically significant differences between P(E) and P(O) were observed for ATP observation (P(O) > P(E); *p*-value = 0.0010), whereas no significant differences were observed for VCC observation (P(O) ≈ P(E); *p*-value = 0.8813). This result indicates that contaminating NP mixtures may have synergistic or at least additive effects on an ecosystem.

The present work demonstrated that mixtures of the tested NPs could show particular modes of action, synergistic or additive effects, depending on the method of biomass measurement. This result indicated that one method of biomass measurement could not provide results equivalent to those of another one. Therefore, combining results (e.g., ATP and VCC measurements) as opposed to just using a single method is a better strategy for the bioassessment of the effects of NPs on microbial growth. Clearly, more work needs to be conducted to understand the mechanism of action of monotypic NPs and NP mixtures in environmental samples. 

## 4. Materials and Methods

### 4.1. Soil and Inoculant Source Characteristics

Soil samples were collected from artificial recharge sites near the Nakdong river in Korea. Five samples from each site were collected, and the mixture was used as a test soil sample. Characteristics of sandy soil are presented in Table 3. Pond water was used as a microbial inoculant source for biofilm formation on the sandy soil. The average characteristics of the pond water (inoculant source) were as follows: T-N 155 mg/L, COD_Mn_ 4.6 mg/L, pH 7.8, and total coliforms 1300 MPN/100 mL. Results of each condition were compared based on relative value of control set.

**Table 3:** Characteristics of the sandy soil used in this study.

Parameter	Value	Soil Particle Size (mm)	Proportion (% wt.)
organic matter (%)	0.30	fine sand (<0.300)	13.2
pH	6.41	medium sand (0.3–1.7)	80.8
bulk density (g/cm^3^)	1.647	coarse sand (>1.7)	6.0
porosity	0.39		

### 4.2. Experimental Setup for Microbial Growth and the Effects of NPs

The optimum conditions for batch microbial growth on sandy soil were determined based on preliminary tests under several conditions, including batch incubation period, inoculant type, nutrients, *etc.* A mixture composed of 20 g of air-dried soil, 5 mL of pond water (inoculation source), and 45 mL of minimum salt medium (MSM) with 0.1% yeast extract was incubated for 30 days at 25 °C and 130 rpm. MSM was composed of 0.2 g of MgSO_4_·7H_2_O, 0.1 g of CaCl_2_, 0.05 mg of FeSO_4_·7H_2_O, 0.25 mg of NaM_O_O_4_·2H_2_O, 0.43 g of K_2_HPO_4_, 0.23 g of KH_2_PO_4_, and 1000 mL of distilled water.

The effects of NPs on microbial growth were individually assessed at concentrations of 0–300 mg/L (ZnO, NiO, and Co_3_O_4_) and 0–1000 mg/L (TiO_2_). The following concentrations, close to the EC_50_ of single NPs based on ATP content, were chosen to investigate the effects of binary NP mixtures on microbial growth: 10 mg/L of ZnO, 70 mg/L of NiO, 30 mg/L of Co_3_O_4_, and 500 mg/L of TiO_2_. After 30 days of incubation, microbial growth on the sandy soil was determined based on ATP content, DHA, and VCC. All other measurements were performed using modified standard methods [51].

### 4.3. Microbial Activity Measurement Based on ATP Content, DHA, and VCC

For ATP (adenosine triphosphate) measurement, 10 g of soil sample after 30 days of incubation was washed twice using a 0.85% NaCl solution. Prewashed sandy soil and 20 mL of sterilized dH_2_O was sonicated for 30 min to extract biomass from it. Distilled water was boiled by heating water on a hot plate, after which 1 mL of boiling water was added to the centrifuged cell pellet and vortexed. Samples were prepared based on manufacture’s protocol (Enliten ATP Assay System, Promega, Madison, WI, USA), ATP content was measured using a Luminometer 20/20 (Turner Design Inc., Sunnyvale, CA, USA).

For DHA measurement, soil samples (3 g) and CaCO_3_ (0.03 g) were mixed by vortexing with 1 mL of 3% TTC (2, 3, 5-triphenyl tetrazolium chloride) (Sigma-Aldrich Co., St. Louis, MO, USA), and distilled water (2.5 mL) was then added and the solution was vortexed again. Following incubation at 37 °C for 24 h, 20 mL of methanol was added and shaken for 20 min [51].

For measurement of VCC, 10 g of soil was mixed with 20 mL of distilled water and sonicated for 5 min. The extracted biomass were serially diluted and spread on a Petri dish containing R_2_A solid medium. Colonies were counted following two days of incubation (25 °C). 

### 4.4. Statistical Analysis and Chemicals

Four types of NPs were tested in this study. Metal oxide NPs of TiO_2_ (<25 nm), NiO (30 nm), and Co_3_O_4_ (10–30 nm) were obtained from Nanostructured and Amorphous Materials (Houston, TX, USA). Metal oxide ZnO (40–100 nm) was obtained from Alfa Aesar (Ward Hill, MA, USA). To prepare stock solutions of NPs, NPs were suspended directly in deionized water and dispersed by ultrasonic vibration for 10 min prior to use. All other chemicals were of reagent grade and purchased from Sigma and Aldrich (St. Louis, MO, USA) or Fisher (Pittsburg, PA, USA). All stock NP solutions were dissolved in sterile distilled water. Following incubation, the metal of the amended NPs in solution was analyzed by inductively coupled plasma-optical emission spectrometry (ICP-OES; Perkin-Elmer Optima 7300DV, Waltham, MA, USA).

The EC_50_ (concentration of a chemical where 50% of its effect is observed) values of single NPs used for microbial growth were determined using the Trimmed Spearman–Karber method [20]. The SPEARMAN computer program (US EPA’s Center for Exposure Assessment Modeling) was used to estimate EC_50_ values. In the mixture test, the theoretically expected effects of the binary mixtures were evaluated using a simple mathematical model based on the theory of probabilities, which has been used before by several researchers [18,52].

P(E) = P_1_ + P_2_ − (P_1_P_2_/100)

P_1_: inhibition caused by chemical “1”; P_2_: inhibition caused by chemical “2”; P(E): theoretically expected inhibition.

The observed inhibition of the binary mixture P(O) determined by the experiment was compared with the theoretically expected inhibition P(E) calculated by the above equation. The mode of interaction was characterized as additive when the difference between P(O) and P(E) was not significant. The mode of interaction was also characterized as synergistic or antagonistic when the observed value was significantly higher or lower, respectively, than the theoretically predicted value at a *p* (null hypothesis) <0.05 level of significance. The 95% level of significance of the results was calculated according to Student’s *t*-test (http://www.graphpad.com).
